# Quantifying spatial and dynamic lung abnormalities with 3D PREFUL FLORET UTE imaging: A feasibility study

**DOI:** 10.1002/mrm.30416

**Published:** 2025-01-17

**Authors:** Filip Klimeš, Joseph W. Plummer, Matthew M. Willmering, Alexander M. Matheson, Abdullah S. Bdaiwi, Marcel Gutberlet, Andreas Voskrebenzev, Marius M. Wernz, Frank Wacker, Jason Woods, Zackary I. Cleveland, Laura L. Walkup, Jens Vogel‐Claussen

**Affiliations:** ^1^ Institute of Diagnostic and Interventional Radiology Hannover Medical School Hannover Germany; ^2^ Biomedical Research in Endstage and Obstructive Lung Disease Hannover German Center for Lung Research Hannover Germany; ^3^ Center for Pulmonary Imaging Research Cincinnati Children's Hospital Medical Center Cincinnati Ohio USA; ^4^ Department of Biomedical Engineering University of Cincinnati Cincinnati Ohio USA; ^5^ Imaging Research Center, Department of Radiology Cincinnati Children's Hospital Medical Center Cincinnati Ohio USA; ^6^ Department of Pediatrics University of Cincinnati College of Medicine Cincinnati Ohio USA; ^7^ Department of Physics University of Cincinnati Cincinnati Ohio USA

**Keywords:** FLORET, hyperpolarized ^129^Xe MRI, lung MRI, motion compensation, PREFUL, UTE, ventilation imaging

## Abstract

**Purpose:**

Pulmonary MRI faces challenges due to low proton density, rapid transverse magnetization decay, and cardiac and respiratory motion. The fermat‐looped orthogonally encoded trajectories (FLORET) sequence addresses these issues with high sampling efficiency, strong signal, and motion robustness, but has not yet been applied to phase‐resolved functional lung (PREFUL) MRI—a contrast‐free method for assessing pulmonary ventilation during free breathing. This study aims to develop a reconstruction pipeline for FLORET UTE, enhancing spatial resolution for three‐dimensional (3D) PREFUL ventilation analysis.

**Methods:**

The FLORET sequence was used to continuously acquire data over 7 ± 2 min in 36 participants, including healthy subjects (*N* = 7) and patients with various pulmonary conditions (*N* = 29). Data were reconstructed into respiratory images using motion‐compensated low‐rank reconstruction, and a 3D PREFUL algorithm was adapted to quantify static and dynamic ventilation surrogates. Image sharpness and signal‐to‐noise ratio were evaluated across different motion states. PREFUL ventilation metrics were compared with static ^129^Xe ventilation MRI.

**Results:**

Optimal image sharpness and accurate ventilation dynamics were achieved using 24 respiratory bins, leading to their use in the study. A strong correlation was found between 3D PREFUL FLORET UTE ventilation defect percentages (VDPs) and ^129^Xe VDPs (r ≥ 0.61, *p* < 0.0001), although PREFUL FLORET static VDPs were significantly higher (mean bias = −10.1%, *p* < 0.0001). In diseased patients, dynamic ventilation parameters showed greater heterogeneity and better alignment with ^129^Xe VDPs.

**Conclusion:**

The proposed reconstruction pipeline for FLORET UTE MRI offers improved spatial resolution and strong correlation with ^129^Xe MRI, enabling dynamic ventilation quantification that may reveal airflow abnormalities in lung disease.

## 1 INTRODUCTION

MRI of the lung is constrained by several challenges, including low proton density, which limits parenchymal signal; rapid transverse magnetization decay, which reduces high‐frequency signal information and increases blurring; and motion artifacts caused by cardiac and respiratory motion.^1^ These challenges can be mitigated by using a sequence that (i) samples from the center of k‐space outward, to oversample low‐frequency signal regions and increase robustness to motion; (ii) has a short echo time, to minimize low‐frequency signal loss; (iii) has short readouts, to minimize T_2_* signal loss in high‐frequency regions of k‐space; and (iv) samples k‐space in a temporally incoherent manner, such that subsequent aliasing caused by retrospective subsampling of data propagates as incoherent noise (e.g., when dividing data into respiratory‐resolved bins).

One sequence that meets all these requirements is three‐dimensional (3D) fermat‐looped orthogonally encoded trajectories (FLORET), which samples 3D volumes using efficient Fermat spirals projected outward from the center of k‐space. Willmering et al. demonstrated that FLORET sampling of the lungs supports high parenchymal signal and increased sharpness compared with 3D radial kooshball, with increased sampling efficiency and thus reduced scan time.^2^, ^3^ Additionally, FLORET can be tuned to sample k‐space in a highly incoherent manner, through the use of multiple hubs and interleave ordering schemes to distribute the spiral arms.^4^ Consequently, we reason that FLORET could be highly suitable for dynamic lung MRI, where continuously acquired data are retrospectively gated according to respiratory position and reconstructed into a series of images throughout the breathing cycle. However, such dynamic lung MRI reconstruction and analysis using FLORET sampling has yet to be explored in the literature.

Recently, a promising reconstruction technique for handling respiratory motion was proposed called motion‐compensated low rank (MoCoLoR) reconstruction.^5^ This approach relies on the assumption that the matrix rank between reconstructed images at different respiratory states, when registered to each other, should be inherently low. Subsequently, the MoCoLoR algorithm converges to a solution that has regularized nuclear norm sparsity along the registered respiratory phase dimension. This approach demonstrated high‐resolution reconstructions of data acquired using 3D radial kooshball sampling, binned into up to 50 respiratory frames. However, this has not yet been applied to FLORET sampling, which may offer improved signal‐to‐noise ratio (SNR), image sharpness, and sampling efficiency.

Following reconstruction, the respiratory‐resolved image reconstructions can be compared against each other to assess pulmonary ventilation.^6^ Numerous publications have focused on this topic, including Fourier decomposition,^7^ SENCEFUL (self‐gated non‐contrast‐enhanced functional lung),^8^ phase‐resolved‐functional lung (PREFUL),^9^ and matrix pencil MRI.^10^ Except for PREFUL or SENCEFUL, the techniques mentioned here have been limited to two‐dimensional (2D) imaging. Three‐dimensional PREFUL MRI has been proposed previously using spoiled gradient‐echo sequence with stack‐of stars acquisition and golden‐angle increment, but has yet to be applied to 3D FLORET sampling where image quality and sampling efficiency are improved.^11^

The objectives of this study were to optimize a binning algorithm and implement MoCoLoR image reconstruction for 3D FLORET ultrashort echo time (UTE) acquisitions. The resultant respiratory‐resolved images undergo a 3D PREFUL algorithm to extract static and dynamic ventilation parameters. The feasibility of the proposed methods was tested, and the static and dynamic ventilation information of 3D PREFUL FLORET UTE were compared with the direct static measurement of pulmonary ventilation derived by ^129^Xe MRI in volunteers with distinct pulmonary disease and healthy controls.

## 2 METHODS

### 2.1 Human participants

Thirty‐six participants were imaged after obtaining informed consent (and age‐appropriate assent for participants aged 11 years and older) following a protocol approved by the Institutional Review Board at Cincinnati Children's Hospital Medical Center (with Food and Drug Administration approval for ^129^Xe MRI IND‐123577). The studied population included healthy participants and participants with a range of lung conditions, including cystic fibrosis (CF), lymphangioleiomyomatosis (LAM), post–hematopoietic stem‐cell transplant recipients (post‐HSCT), bronchiolitis obliterans syndrome (BOS), and neuroendocrine cell hyperplasia of infancy. Table 1 describes the study participant demographics.

**TABLE 1:** Study participants characteristics and quantitative ventilation measurements derived using the ^129^Xe and 3D PREFUL FLORET MRI.

	All study participants (*N* = 36)	Healthy participants (*N* = 7)	CF (*N* = 14)	LAM (N = 7)	Post‐HSCT (*N* = 4)	BOS (*N* = 3)	NEHI (*N* = 1)	*r* Value^a^
Age range	7–55	8–27	7–22	39–55	11–21	14–21	13	‐
Sex	21 M/15 F	5 M/2 F	8 M/6 F	7 F	4 M	3 M	1 M	–
VDP_Xe_ [%]	7.1 (4.6–14.9)	4.2 (3.1–4.5)	14.9 (4.4–21.3)	8.1 (6.6–10.9)	5.9 (5.4–7.1)	11.8 (8.3–16.9)	14.1	–
VDP_RVent_ [%]	18.6 (12.7–25.5)	10.8 (6.7–11.5)	18.9 (14.9–31.4)	21.4 (19.1–29.8)	16.0 (14.3–18.0)	17.7 (16.1–29.9)	25.1	0.76*
VDP_FVL‐CM_ [%]	10.4 (4.1–15.5)	1.9 (1.4–4.6)	8.9 (7.2–16.1)	13.6 (11.7–14.9)	10.0 (6.1–12.4)	12.6 (7.4–21.7)	20.5	0.61*
Mean VTTP [%]	50.7 (48.1–53.4)	49.1 (47.5–51.8)	50.3 (49.4–51.9)	51.7 (49.3–53.7)	45.8 (43.7–49.3)	54.0 (52.4–54.7)	56.0	0.36*

*Note*: Values of ventilation parameters are median with interquartile range in brackets.

Abbreviations: 3D, three‐dimensional; BOS, bronchiolitis obliterans syndrome; CF, cystic fibrosis; F, female; HSCT, hematopoietic stem‐cell transplant; LAM, lymphangioleiomyomatosis; M, male; NEHI, neuroendocrine cell hyperplasia of infancy; Post‐HSCT, post–hematopoietic stem cell transplantation; PREFUL, phase‐resolved functional lung; VDP_FVL‐CM_, ventilation defect percentage based on flow‐volume‐loop correlation metric; VDP_RVent_, VDP based on regional ventilation; VDP_Xe_, VDP based on the Xenon; VTTP, ventilation time‐to‐peak.

^a^ Pearson correlation analysis (*r*) assessed the relationship between ventilation parameters derived using the ^129^Xe (VDP_Xe_) and 3D PREFUL FLORET UTE MRI for all study participants.

* Significant correlations: *p* < 0.05.

### 2.2 Image acquisition– ^129^Xe

#### 2.2.1 Acquisition and reconstruction

Xenon gas (enriched to > 85% ^129^Xe) was hyperpolarized to 20%–40% using a continuous‐flow, Polarean 9820A ^129^Xe hyperpolarizer (Polarean Imaging PLC, Durham, NC, USA).^12^ Polarized gas was administered to patients from volume‐matched Tedlar bags (Jensen Inert, Coral Springs, FL, USA). ^129^Xe gas was dosed at one‐sixth of the subject sex‐predicted and height‐ predicted total lung capacity (up to 1 L) for CF patients,^13^ and one‐sixth of the predicted forced vital capacity (up to 1 L) for the remaining study participants. Doses were administered from expiration at functional residual capacity.

Breath‐hold (< 15 s) ^129^Xe ventilation images were acquired on a 3T Philips Ingenia‐Elition MRI scanner (Philips Healthcare, Best, Netherlands). ^129^Xe acquisitions were performed with a quadrature vest coil (Clinical MR Solutions, Brookfield, WI, USA). Images were acquired with a multislice 2D spoiled gradient‐echo sequence, field of view (FOV_xy_) = (300–400)mm^2^, FOV_z_ = 150–200 mm, resolution = (3 × 3 × 15)mm^3^, slice gap = 0 mm, echo time (TE)/repetition time (TR) = < 5 ms/< 10 ms, and total scan duration < 15 s. ^129^Xe calibration scans were performed to calculate the optimal flip angle (8°–20°), depending on the number of excitations and receiver frequency for each subject. All scans were completed within < 60‐min total time, with ^129^Xe scans performed first, and FLORET UTE scans performed last (starting at about 45 min).

#### 2.2.2 VDP analysis

^129^Xe ventilation defect percentage (VDP_Xe_) was calculated from the percentage of the lung voxels with signal intensity below 60% of the mean whole‐lung signal intensity.^14^, ^15^ Image segmentations were performed using an in‐house pretrained deep learning model that excludes the airways.^16^ Ventilation images were corrected for B_1_ inhomogeneity using N4‐ITK bias correction before VDP calculation.^17^

### 2.3 Image acquisition–proton

#### 2.3.1 Acquisition

Proton lung images were acquired during free breathing using a 3D FLORET UTE sequence, with the following acquisition parameters: FOV = (320 ± 40 mm)^3^, acquisition resolution = (1.6 mm)^3^, TE/TR = 0.1/3.76 ms, readout length = 0.95 ms, flip angle = 4.0°, total excitations = 140 000 ± 20 000, hubs = 3, total scan duration = 7 ± 2 min. The variable number of excitations were a result of controlling the Nyquist sampling percentage for each exam's FOV. Images were acquired with a 16‐channel anterior coil and a 12‐channel posterior coil with a respiratory bellows belt to acquire respiratory position with each excitation. Images were acquired within 30 min of the ^129^Xe ventilation acquisition.

#### 2.3.2 Binning algorithm

**FIGURE 1:**
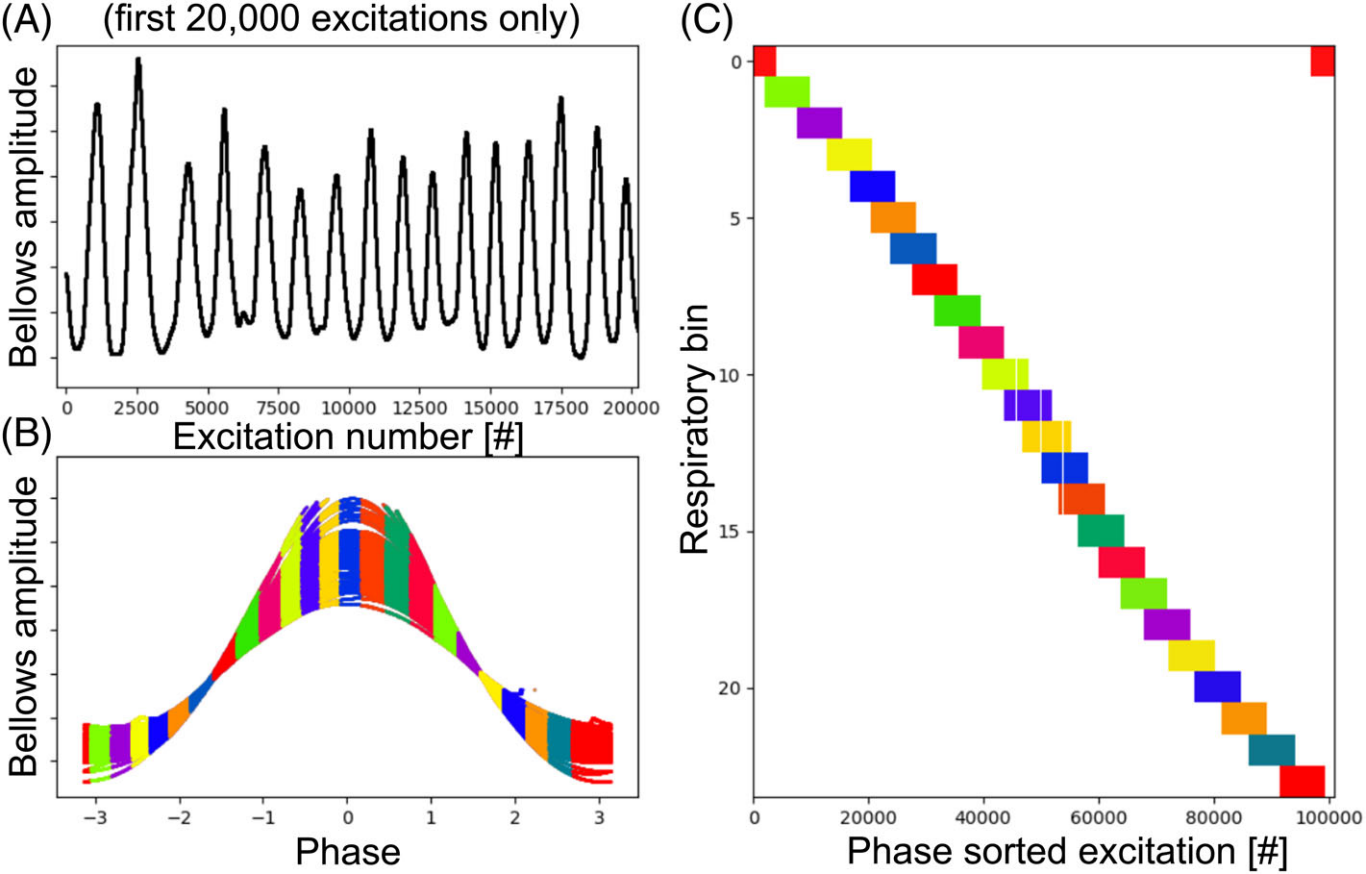
Respiratory binning workflow. (A) Waveform collected from respiratory bellows measurement (only first 20 000 excitations; ˜1 min shown). (B) Hilbert‐transform of respiratory bellows waveform to separate each respiratory cycle into phase position −π to π. Transformed data were binned into 24 windows containing 10 000 excitations each. Different colors refer to different respiratory bins. (C) The degree of overlap within each bin. Small white lines in (C) correspond to excluded excitations that fell beyond ±2 standard deviations of the mean respiratory bellows measurement.

The binning algorithm described by Capaldi et al.^18^ was used and optimized in this study, as illustrated in Figure 1. First, the respiratory bellows signal was smoothed and filtered using a moving average function. Second, high‐amplitude and low‐amplitude oscillations were excluded (±2 standard deviations of the mean bellows reading). Third, the bellows reading indices (corresponding to each RF excitation) were sorted into [8, 12, 16, 20, 24, 32, 40] respiratory‐motion bins according to instantaneous phase calculated by Hilbert transformation and applied to raw k‐space data and trajectories. Equal numbers of excitations were sorted into each respiratory motion bin ([30 000, 20 000, 15 000, 12 000, 10 000, 7500, 6000] excitations per bin), such that the total amount of excitations equaled 240 000. Dynamic view sharing was permitted such that each respiratory bin shared up to 50% excitations.

#### 2.3.3 Reconstruction

**FIGURE 2:**
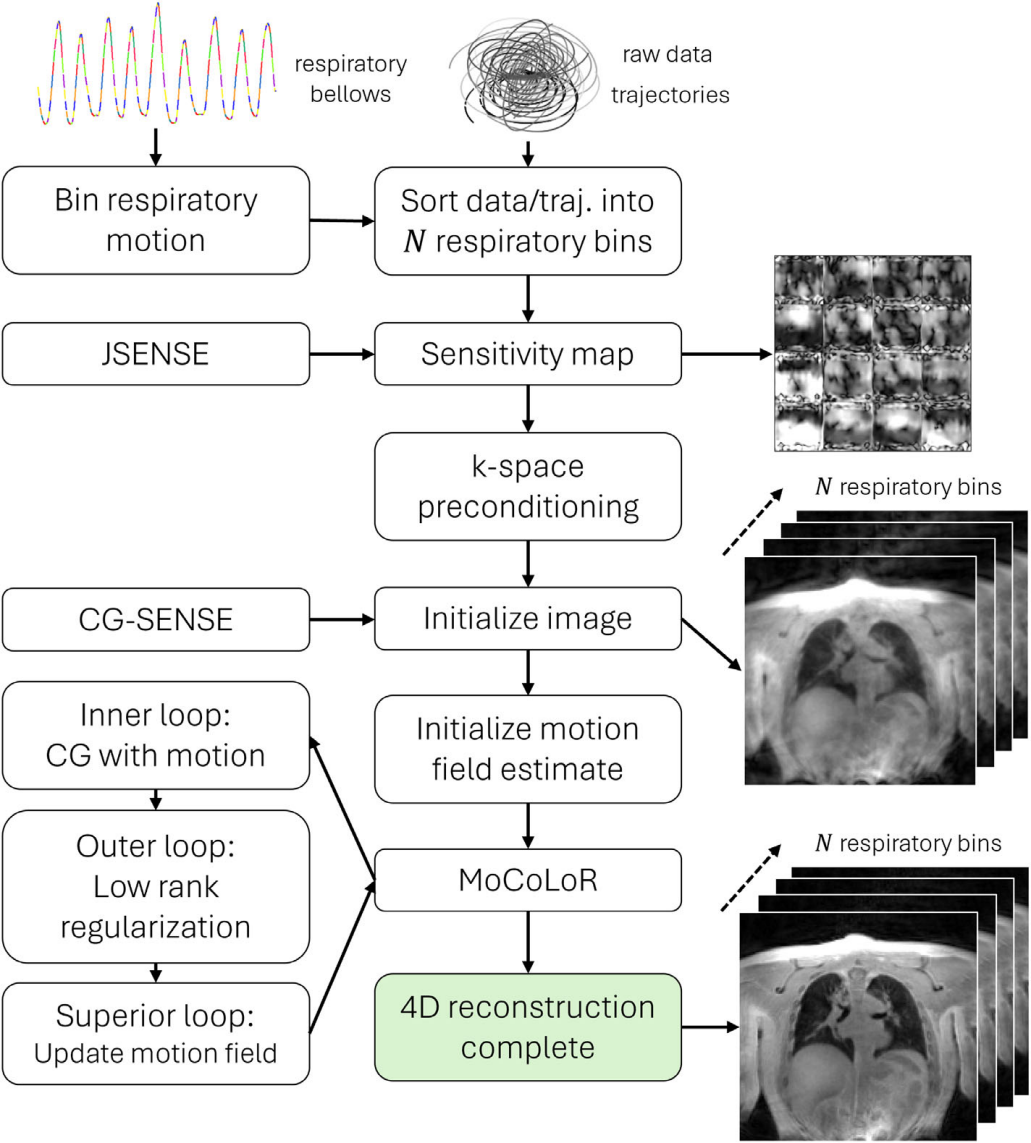
Three‐dimensional FLORET (fermat‐looped orthogonally encoded trajectories) reconstruction pipeline for this study. In chronological order: (1) Excitation indices are binned according to position in respiratory cycle and applied to raw k‐space data and trajectories; (2) sensitivity maps and k‐space preconditioners are calculated; (3) conjugate‐gradient sensitivity encoding (CG‐SENSE) is explored to initialize N images corresponding to each respiratory phase; (4) motion field from initial images is estimated, iterated through the motion‐compensated low‐rank reconstruction (MoCoLoR) algorithm using the initial motion fields as inputs; and (5) reconstruction is complete.

Proton data were reconstructed with multiple respiratory phases using a motion‐compensated low rank (MoCoLoR) reconstruction algorithm.^5^ The following reconstruction problem was solved:

(1) $$x=\underset{x}{\text{argmin}}{\frac{1}{2}\left\|P\left(\mathrm{FSx}-y\right)\right\|}_{2}^{2}+\lambda_{L}{\left\|\mathrm{Mx}\right\|}_{*},$$

where $x\in {\mathbb{C}}^{B\times N^{3}}$ is the underlying image with B respiratory states and $N^{3}$ image size; $P\in {\mathbb{R}}^{B\times I\times J}$ are the k‐space preconditioning weights for I excitations (readouts) per respiratory phase and J samples per readout; $F\in {\mathbb{C}}^{B\times I\times J}$ is the nonuniform fast Fourier transform; $S\in {\mathbb{C}}^{C\times N^{3}}$ are the coil sensitivity maps for C coils; $y\in {\mathbb{C}}^{B\times C\times I\times J}$ are the binned k‐space measurements; λ_L_ is the low‐rank regularization parameter; ${\left\|\cdot \right\|}_{*}$ is the nuclear norm; and $M\in {\mathbb{R}}^{J}$ are the motion field operators. P were calculated using the single‐channel k‐space preconditioning approach by Ong et al., as it offered the best balance between computation time and image quality.^19^ Motion field operators were estimated using registration software within the ANTs framework.^20^ Sensitivity maps were estimated using the JSENSE/NLINV algorithm described by Uecker et al.^21^ and Ying et al.^22^ The full reconstruction workflow is visualized in Figure 2. Reconstructions were performed in *Python* 3.12, using linear algebra functionality from SigPy, and an NVIDIA‐A5000 (24GB) graphical processing unit on a Linux distribution. Reconstruction settings included resolution = (3 × 3 × 3)mm^3^, λ_L_ = 0.01, inner iterations = 5, outer iterations = 3, superior iterations = 3, and step size ρ= 1.

#### 2.3.4 PREFUL analysis

##### 2.3.4.1. Image registration and postprocessing

Following image reconstruction, the respiratory‐resolved data were resorted such that the ventilation cycle started at end‐expiration, running to end‐inspiration, and ending back at end‐expiration. Subsequently, the reordered respiratory‐resolved images underwent bias‐field correction using the “N4BiasFieldCorrection” algorithm,^17^ integrated within ANTs framework to alleviate intensity inhomogeneity.

To enable voxel‐wise analysis of the respiratory‐resolved data, it is necessary to implement motion‐compensation techniques to account for the movement of the heart and lungs. Consequently, following a method described by Klimeš et al.,^11^ all respiratory phases were subsequently stepwise‐registered to the phase at maximum inspiration using the ANTs toolkit. During registration, the initial affine transform was followed by the nonrigid flexible registration. For the nonrigid registration, a B‐spline symmetric normalization diffeomorphic registration algorithm was executed with the following parameters: gradient step = 1, updated field mesh size at base level = 40, total field mesh size at base level = 0, spline order = 3, metric = cross‐correlation, radius = 4, metric weight = 1, convergence parameter = 150 × 150 × 100 × 80/1 × 10^−6^/10, shrink parameters = 6 × 4 × 2 × 1, and smoothing parameters = 3 × 2 × 1 × 0. For both rigid and nonrigid transformations, a rectangular mask covering the entire 3D lung volume was used to reduce convergence time.

Finally, the respiratory images were interpolated into 16 equidistant phases using a Gaussian kernel with a sigma value of 0.3, inspired by the approach outlined by Voskrebenzev et al.^9^ To mitigate signal variations unrelated to respiratory motion, a low‐pass filter with a cutoff frequency of 0.7 Hz was used on the interpolated phases. Additionally, a 3D edge‐preserving filter was applied to the images to maintain the structural integrity while removing noise.^23^

##### 2.3.4.2. Lung parenchyma and vessel segmentation

The lung cavity within the end‐inspiration image was used as a reference image for image registration. The cavity was segmented using an in‐house pretrained deep learning model^16^ that excludes the airways and subsequent manual refinement by a postdoctoral researcher with 8 years of experience in pulmonary MRI. The lung‐cavity segmentation was followed by a lung‐vessel recognition.^24^ The final lung parenchyma mask was generated by subtracting the lung‐vessel mask from the lung‐cavity mask.

##### 2.3.4.3. Ventilation parameters

The ventilation parameters used in this study were based on those outlined in the original 3D PREFUL publication by Klimeš et al.^11^ Regional ventilation (RVent) was quantified for each respiratory phase of the entire ventilation cycle.^25^ RVent, a static ventilation parameter, considers two respiratory phases in its calculation and has been demonstrated to be influenced by both respiratory frequency and depth.^26^, ^27^ The RVent map of the end‐inspiration phase was then further considered for the image and statistical analysis.

The RVent cycle within each voxel was evaluated using flow‐volume loops (FVLs), where flow (the first derivative of RVent) was plotted on the y‐axis and volume (RVent) on the x‐axis. To establish a baseline, a high‐ventilation reference FVL was generated from a region where RVent values fall between the 75%–95% quantiles. The similarity between the FVL of each voxel and the high‐ventilation reference was then evaluated using the cross‐correlation metric (CM).

For both the RVent at end‐inspiration and the FVL‐CM parameters, maps depicting ventilation defects and VDP values were calculated using established thresholds.^9^, ^28^ Although a fixed threshold of 0.9 was used for FVL‐CM, resulting in VDP_FVL‐CM_ values, RVent used a variable threshold. Specifically, the threshold for RVent was determined as 40% of the 90th percentile value, resulting in VDP_RVent_ values. Both VDP parameters, derived using those published thresholds, have demonstrated a strong correlation with spirometry measurements,^11^ ^129^Xe ventilation maps,^29^ and more recently, with VDP values obtained through direct visualization of ventilation using ^19^F MRI.^30^

Subsequently, ventilation time‐to‐peak (VTTP) maps as a percentage of the ventilation cycle were generated. VTTP denotes the time at which ventilation attains its maximal magnitude within the whole ventilation cycle. In regions exhibiting normal physiology, ventilation ideally achieves its peak at approximately 50% of the cycle duration. Prolonged VTTP values may signify diminished or delayed ventilatory dynamics.

### 2.4 Statistical analysis

To establish the optimal regularization settings, a range of λ_L_ parameters (0.005–0.025) were used for MoCoLoR reconstruction. The quality of the ventilation images obtained from 3D PREFUL FLORET UTE was assessed both visually and quantitatively with Sobel image sharpness (∇), using data from a single healthy volunteer.

Postprocessing optimization was conducted in a subcohort consisting of 10 study participants (5 healthy participants, 2 patients with CF, 2 patients with BOS, and 1 patient with LAM). The 3D PREFUL analysis was assessed across different respiratory binning settings, including [8, 12, 16, 20, 24, 32, 40] respiratory bins. Significance testing using Kruskal‐Wallis analysis was performed to evaluate differences in ventilation parameters (mean RVent, standard deviation RVent, mean FVL‐CM, standard deviation FVL‐CM, mean VTTP, VDP_RVent_, and VDP_FVL‐CM_), as well as in signal‐to‐noise ratio (SNR) and mean sharpness of the RVent images across all settings. SNR was calculated in the central coronal slice at the level of the trachea bifurcation of a morphological image used as a fixed image for registration. At this location, regions of interest were selected inside the lung‐parenchyma mask and in the background. The same regions of interest were used for all tested reconstruction settings. The image sharpness was evaluated using the Sobel gradient operator in all three spatial directions. The final sharpness value was defined as an average gradient magnitude inside the lung‐parenchyma mask.

Subsequently Pearson correlation analysis was used to assess relationships between static VDP_Xe_ with static VDP_RVent_ and dynamic VDP_FVL‐CM_ in the whole study cohort, and the mean differences of the VDP values were quantified by Bland–Altman analysis.

Statistical analyses were performed using *JMP Pro* 17 (SAS Institute, Cary, NC, USA), and the level of statistical significance was set at *p* < 0.05.

## 3 RESULTS

### 3.1 Image‐reconstruction optimization

**FIGURE 3:**
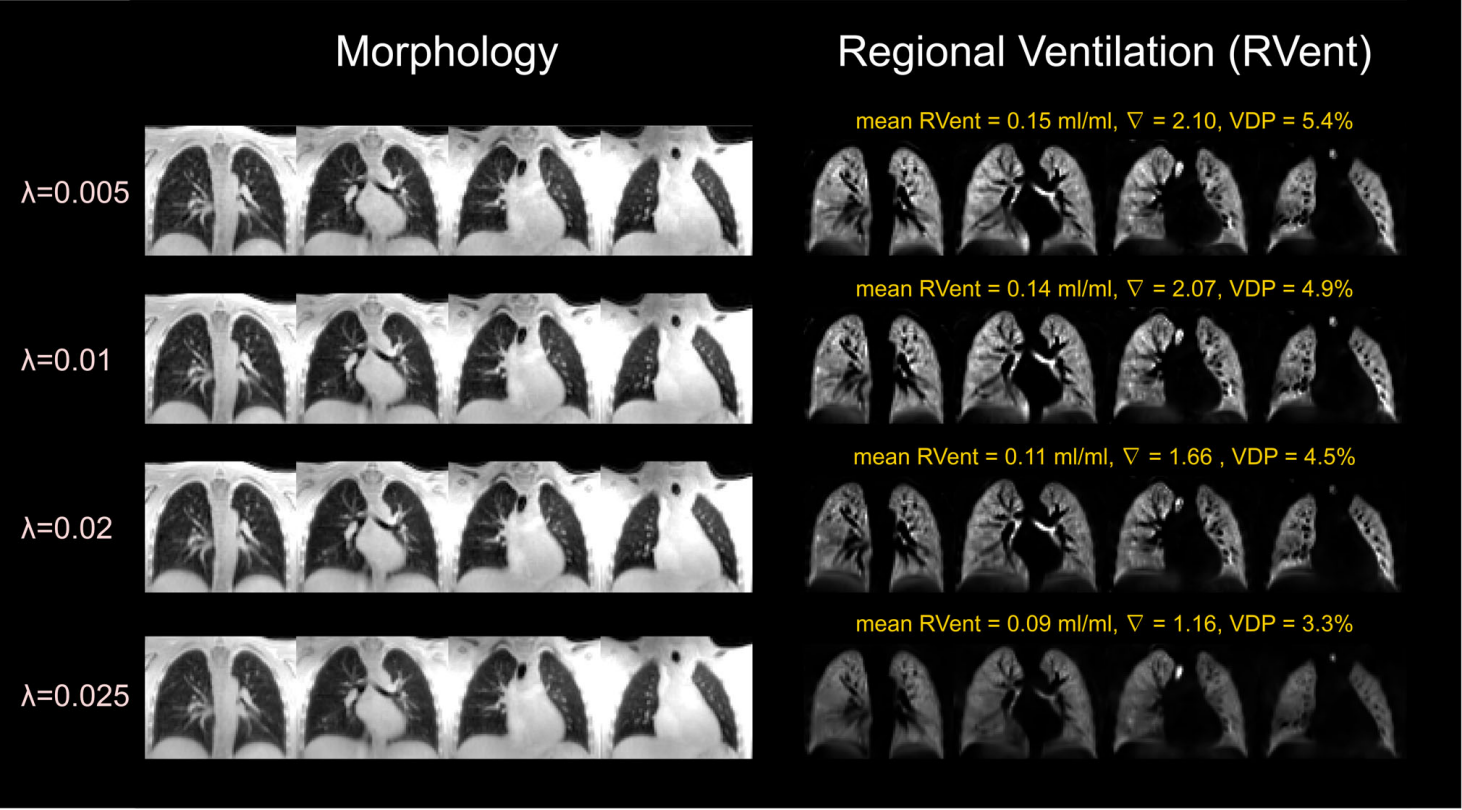
Comparison of different regularization parameters and their influence on image quality of ventilation images derived from three‐dimensional PREFUL (phase‐resolved functional lung) FLORET (fermat‐looped orthogonally encoded trajectories) ultrashort echo time. Overview of morphological end‐expiration (*left*) and regional ventilation (RVent; *right*) maps derived using different reconstruction settings (regularization parameter, λ_L_) for a healthy participant (male, 11 years old). Note the superior Sobel image sharpness (∇) of ventilation images and improved qualitative sharpness around diaphragm as well as well‐defined vessels of morphological images for image reconstructions with λ_L_ = 0.005 and 0.01. VDP, ventilation defect percentage.

Morphological end‐inspiration and static 3D PREFUL FLORET UTE ventilation images from the MoCoLoR reconstruction optimizations are shown in Figure 3. The λ_L_ parameters ranging from 0.005 to 0.025 were used for the dynamic MoCoLoR reconstruction. Using Figure 3 as a reference for the rest of the study, image reconstructions with λ_L_ = 0.01 were chosen, as they demonstrated the best balance between image sharpness of ventilation maps (∇ = 2.07 in the Figure 3) and mitigation of aliasing artifacts/noise. Visually examining the morphological end‐inspiration images, the λ_L_ = 0.01 and λ_L_ = 0.005 settings also showed pronounced sharpness in the diaphragm region as well as in the pulmonary vasculature, compared with larger λ_L_ values.

### 3.2 Postprocessing optimization

Regardless of the number of respiratory bins, there were no significant differences in ventilation and image sharpness and SNR parameters across all evaluated reconstruction settings (Table 2; all *p* > 0.87 for all parameters). Despite being statistically insignificant, we observed a small decreasing trend in mean RVent values with increasing number of respiratory bins, ranging from 0.09 mL/mL for 8 bins to 0.07 mL/mL for 40 bins. However, standard deviations of RVent, mean FVL‐CM, standard deviations of FVL‐CM, VDP_RVent_, and VDP_FVL‐CM_ values remained stable without any discernible trend. Additionally, we observed a small but statistically insignificant decrease in mean VTTP values with the increasing number of reconstructed bins, from 52.7% for 8 bins to 49.6% for 40 bins. We observed a statistically insignificant increase in SNR values with the number of reconstructed bins, from 14.0 for 8 bins to 14.9 for 40 bins, while image sharpness decreased slightly from 1.3 to 1.0 between the 8‐bin and 40‐bin settings.

**TABLE 2:** Assessment of ventilation parameters depending on the applied setting for image reconstruction in the study subcohort consisting of 10 participants.

Parameter/setting	8bin	12bin	16bin	20bin	24bin	32bin	40bin	*p*‐Value/chi square^a^
mean RVent [mL/mL]	0.09 (0.06–0.13)	0.09 (0.07–0.14)	0.09 (0.07–0.14)	0.08 (0.06–0.14)	0.08 (0.07–0.14)	0.08 (0.06–0.13)	0.07 (0.06–0.13)	0.96/1.55
std RVent [mL/mL]	0.04 (0.03–0.06)	0.05 (0.03–0.06)	0.05 (0.02–0.06)	0.05 (0.02–0.06)	0.05 (0.03–0.06)	0.05 (0.02–0.06)	0.05 (0.02–0.06)	0.99/1.01
mean FVL‐CM [−]	0.98 (0.96–0.99)	0.98 (0.96–0.99)	0.98 (0.96–0.99)	0.98 (0.96–0.99)	0.98 (0.97–0.99)	0.98 (0.96–0.99)	0.98 (0.97–0.99)	1.00/0.29
std FVL‐CM [−]	0.10 (0.07–0.20)	0.08 (0.07–0.19)	0.09 (0.06–0.20)	0.09 (0.07–0.18)	0.09 (0.06–0.18)	0.11 (0.06–0.20)	0.10 (0.06–0.18)	1.00/0.38
mean VTTP [%]	52.68 (48.18–53.32)	52.05 (48.31–53.17)	51.30 (49.46–52.38)	51.36 (48.08–53.16)	50.87 (49.42–53.61)	49.18 (48.59–51.97)	49.60 (47.64–52.66)	0.98/1.09
VDP_RVent_ [%]	14.02 (10.24–19.29)	13.70 (9.47–16.37)	14.39 (10.27–14.94)	13.52 (9.48–15.11)	13.89 (9.06–14.11)	13.74 (9.59–15.22)	12.61 (9.29–15.46)	1.00/0.36
VDP_FVL‐CM_[%]	2.50 (1.40–4.29)	2.22 (1.48–4.29)	2.50 (1.38–4.67)	2.39 (1.86–4.69)	2.45 (1.80–5.23)	2.98 (1.65–5.14)	2.74 (1.23–4.38)	1.00/0.40
SNR [−]	14.04 (9.66–17.68)	14.85 (9.85–17.31)	13.54 (8.50–19.26)	14.35 (9.12–17.62)	14.52 (7.99–18.15)	15.00 (10.00–20.14)	14.90 (10.70–22.22)	0.91/2.14
Sharpness [−]	1.26 (0.90–1.74)	1.22 (1.01–1.81)	1.17 (1.02–1.79)	1.14 (0.98–1.79)	1.13 (1.00–1.74)	1.05 (0.94–1.64)	0.99 (0.88–1.61)	0.87/2.51

*Note*: Values are median with interquartile range in brackets.

Abbreviations: FVL‐CM, flow volume loop correlation metric; NEHI, neuroendocrine cell hyperplasia of infancy; RVent, regional ventilation; SNR, signal‐to‐noise ratio; std, standard deviation; VDP, ventilation defect percentage; VTTP, ventilation‐time‐to‐peak.

^a^ Kruskal‐Wallis test was used to assess differences for all parameters across different settings. *p*‐Value < 0.05 was deemed significant.

### 3.3 Comparison of ventilation parameters derived using the 3D PREFUL FLORET UTE and ^129^Xe MRI

**FIGURE 4:**
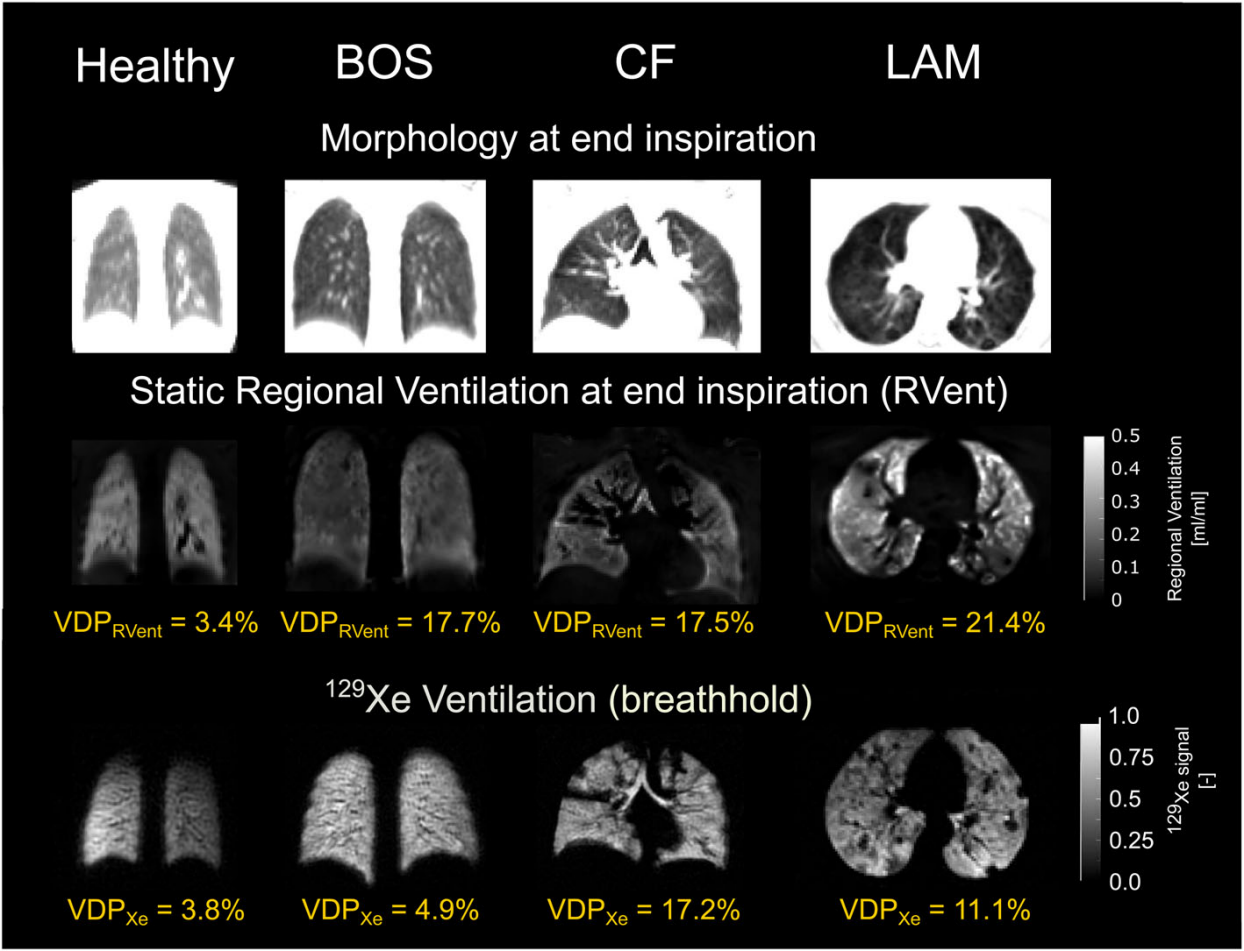
Comparison of static three‐dimensional PREFUL (phase‐resolved functional lung) FLORET (fermat‐looped orthogonally encoded trajectories) ultrashort echo time MRI (*first–second row*) and breath‐hold ^129^Xe ventilation MRI (*third row*). Four study participants are depicted: healthy volunteer (*first column*, female, 13 years old), subject with bronchiolitis obliterans syndrome (BOS; *second column*, male, 24 years old), subject with cystic fibrosis (CF; *third column*, female, 25 years old), and subject with lymphangioleiomyomatosis disease (LAM; *fourth column*, female, 39 years old). For both measurements, the whole‐lung ventilation defect percentage (VDP) values are listed below the respective ventilation map.

Example results demonstrating the correspondence of 3D PREFUL FLORET UTE‐derived static RVent ventilation maps (second row) with ^129^Xe MRI (third row) are shown in Figure 4. Note the homogeneous distribution of ventilation maps for the healthy‐volunteer participant in the first column. In contrast, ventilation maps for patients with disease (second‐fourth columns) depict ventilation heterogeneities for both techniques with good visual overlap.

**FIGURE 5:**
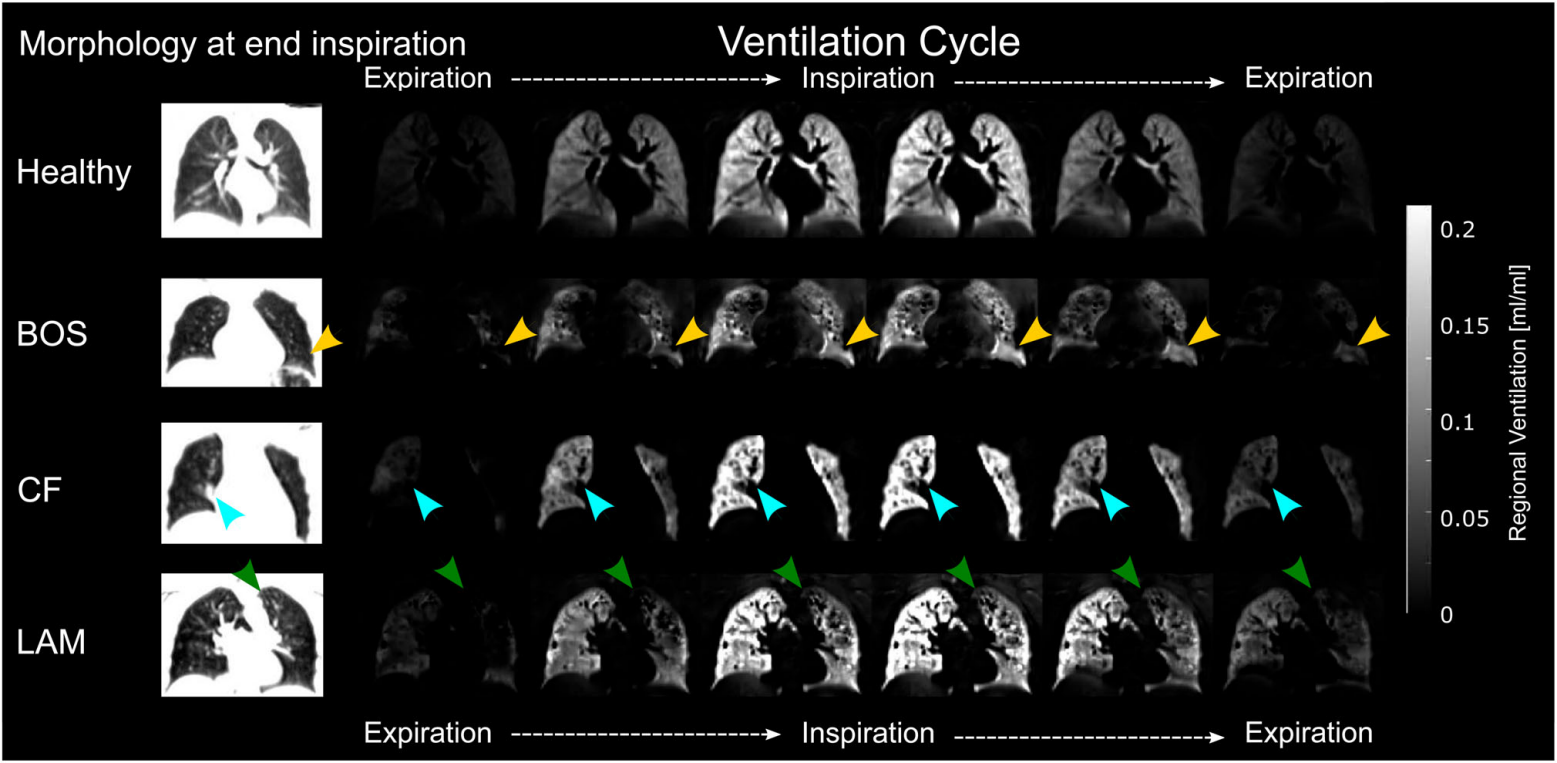
Comparison of three‐dimensional PREFUL (phase‐resolved functional lung) FLORET (fermat‐looped orthogonally encoded trajectories) ultrashort echo time MRI–derived ventilation cycle. Healthy volunteer (*first row*, male, 13 years old), cystic fibrosis (CF; second row, female, 25 years old) patient, bronchiolitis obliterans syndrome (BOS; *third row*, male, 24 years old) patient, and lymphangioleiomyomatosis (LAM; *fourth row*, female, 39 years old) patient are presented. Note homogenous ventilation cycle for the healthy volunteer and no ventilation filling for consolidation in the right lung of the CF patient (*cyan arrows*). For both BOS and LAM subjects, the ventilation dynamics are much more heterogeneous. Dynamic filling asynchronization is indicated with yellow arrows for BOS and green arrows for LAM subjects, suggesting abnormal airflow related to airway narrowing.

Dynamic ventilation cycles of 3D PREFUL FLORET UTE are depicted in Figure 5. A homogeneous ventilation cycle can be observed in the healthy volunteer (first row), whereas abnormal ventilation dynamics were evident in subjects with CF (second row), with BOS (third row) and with LAM (fourth row). In these diseases, airway narrowing may cause abnormal airflow, which is depicted as irregular filling throughout the ventilation cycle.

**FIGURE 6:**
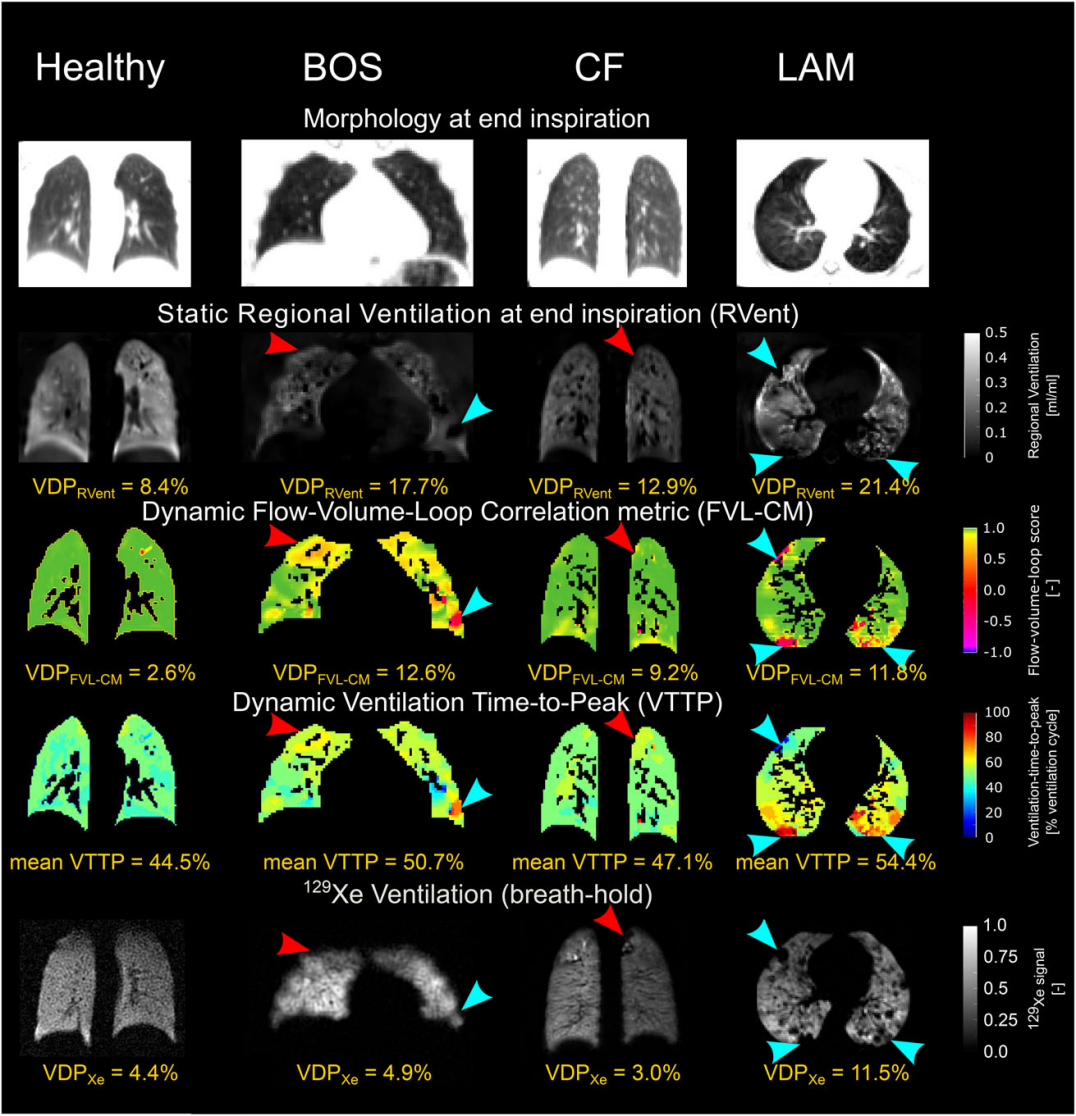
Comparison of static and dynamic three‐dimensional (3D) PREFUL (phase‐resolved functional lung) FLORET (fermat‐looped orthogonally encoded trajectories) ultrashort echo time (UTE) MRI (*first–fourth row*) and breath‐hold ^129^Xe ventilation MRI (*fifth row*). Healthy volunteer (*first column*; male, 25 years old) subject with bronchiolitis obliterans syndrome (BOS; *second column*, male, 24 years old), subject with cystic fibrosis (CF; *third column*, male, 25 years old), and subject with lymphangioleiomyomatosis disease (LAM; *fourth column*, female, 39 years old) are depicted. The overlapping hypoventilation areas depicted in static regional ventilation of 3D PREFUL FLORET UTE (*second row*) and ^129^Xe ventilation imaging (*fifth row*) are marked with cyan arrows. In contrast, overlapping ventilation abnormalities derived with dynamic 3D PREFUL FLORET UTE parameters (*third–fourth row*) and ^129^Xe breath‐hold imaging are marked with red arrows. CM, cross‐correlation metric; FVL, flow‐volume loop; RVent, regional ventilation; VDP, ventilation defect percentage; VTTP, ventilation time‐to‐peak.

Figure 6 presents a comparison of static and dynamic ventilation parameters of 3D PREFUL FLORET UTE and ^129^Xe ventilation images. Healthy subject (first column) showed homogeneous ventilation maps for both techniques. Significant findings were observed in BOS (second column) and CF (third column) patients, wherein dynamic ventilation parameters of 3D PREFUL FLORET UTE (FVL‐CM (third row) and VTTP (fourth row)) exhibit greater heterogeneity compared with the static RVent parameter (second row), aligning better with ^129^Xe ventilation MRI (fifth row) results. For the LAM patient (fourth column), the ventilation defects visible on the ^129^Xe ventilation maps were well‐matched using both the static and dynamic ventilation maps derived from 3D PREFUL FLORET UTE.

A summary of quantitative ventilation measurements for all study participants and participants grouped by disease is shown in Table 1.

**FIGURE 7:**
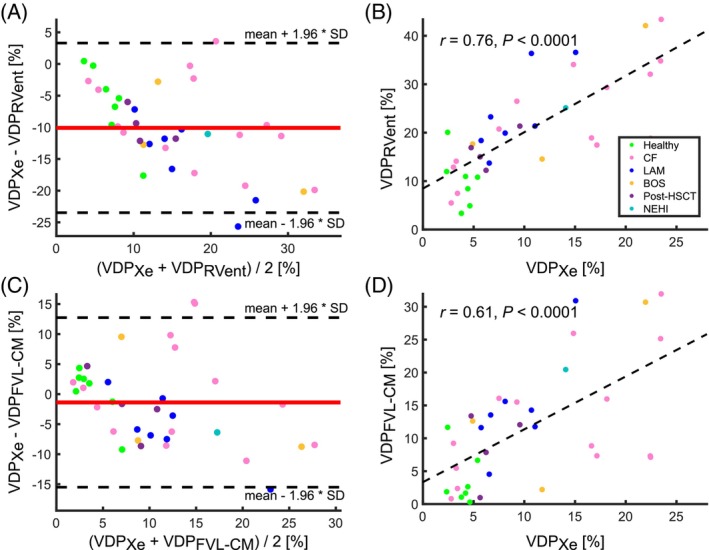
Correspondence of ventilation defect percentage (VDP) values derived by three‐dimensional (3D) PREFUL (phase‐resolved functional lung) FLORET (fermat‐looped orthogonally encoded trajectories) ultrashort echo time MRI and ^129^Xe imaging. Bland–Altman (A,C) and correlation analysis (B,D) of global VDP values derived using 3D PREFUL MRI postprocessing and ^129^Xe MRI. Study groups are visually marked with distinct colors (*green*, healthy volunteers; *pink*, patients with cystic fibrosis [CF]; *blue*, patients with lymphangioleiomyomatosis [LAM]; *orange*, patients with bronchiolitis obliterans syndrome [BOS]; *purple*, patients post–hematopoietic stem cell transplantation [Post‐HSCT]; *cyan*, patient with neuroendocrine cell hyperplasia of infancy [NEHI]). cross‐correlation metric; FVL, flow‐volume loop.

All 3D PREFUL FLORET UTE MRI‐derived VDP values showed significant strong correlations with ^129^Xe MRI VDP values (all r ≥ 0.61, all *p* < 0.0001; Table 1). The static VDP_RVent_ exhibited a stronger correlation with VDP_Xe_ than the dynamic VDP_FVL‐CM_. For VDP_RVent_, the Bland–Altman analysis showed significant differences from VDP_Xe_ (mean bias of −10.1%, *p* < 0.0001; Figure 7A). The correlation plot showing the relationship between static VDP_RVent_ and VDP_Xe_ is displayed in Figure 7B (r = 0.76, *p* < 0.0001). No significant bias was observed between VDP_FVL‐CM_ and VDP_Xe_ (mean bias of −1.4%, *p* = 0.22; Figure 7C). Figure 7D displays the correlation plot showing the relationship between dynamic VDP_FVL‐CM_ and VDP_Xe_ (r = 0.61, *p* < 0.0001). Moderate correlation was found between VTTP and VDP_Xe_ (r = 0.36, *p* = 0.03; Table 1).

## 4 DISCUSSION

This study introduced an optimized image reconstruction pipeline specifically developed for ^1^H FLORET UTE acquisition, providing a series of temporally resolved lung images suitable for pulmonary ventilation assessment with 3D PREFUL. The proposed method offered ventilation images with improved spatial resolution compared with previously published 3D PREFUL results (proposed 3mm^3^ vs. previous 3.9mm^3^)^11^ and demonstrated a strong global correlation with static ^129^Xe MRI. Furthermore, the pipeline enabled calculation of dynamic ventilation parameters, which could be useful for understanding dynamic lung abnormalities caused by respiratory disease.

The first objective of the study was to optimize the respiratory binning (i.e., the number of respiratory phases to include) and MoCoLoR reconstruction settings for subsequent 3D PREFUL analysis. First, we noticed that the PREFUL‐derived VDP did not vary significantly with the number of bins used in the reconstruction. However, despite being statistically insignificant, we noticed that the image sharpness had a small decreasing trend with the increasing number of bins. Second, the increasing number of bins increased the load on the GPU and reconstruction time. Therefore, we opted to use 24 respiratory bins with 10 000 readouts per bin, as a qualitatively optimal trade‐off between image sharpness and temporal resolution of the ventilation cycle. Additionally, a MoCoLoR reconstruction regularization parameter (λ_L_) setting of 0.01 offered a good balance between image sharpness and dynamic signal resolution between respiratory phases for 24 respiratory phases—of which are both highly important when studying heterogeneous abnormalities in the lung. However, it is noted that this binning algorithm and reconstruction approach relies on the quality of the respiratory bellows signal. Subsequently, detection of bulk motion using this method may not be feasible. Alternatively, sorting based on the zeroth k‐space sampling point (DC‐gating)^31^ or use of a convolutional neuronal network for motion compensation^32^ could enhance the stability of this binning algorithm. Further sorting according to cardiac phase may improve the accuracy of ventilation mapping in regions strongly affected by cardiac motion during free‐breathing acquisition.

By using the proposed MoCoLoR reconstruction scheme and applying the 3D PREFUL postprocessing algorithm, ventilation analysis was feasible for all study participants. In contrast to current 3D PREFUL literature,^11^ this study achieved improved spatial resolution of 3 mm (isotropic) compared with the original publication's 3.9 mm (isotropic), potentially enhancing sensitivity to lung‐ventilation abnormalities. Notably, it is possible to obtain higher‐resolution images, as data were acquired at 1.8 mm; however, the reconstruction and registration times during image processing would have taken considerably longer to handle the larger matrix size. Consequently, 3 mm was chosen as the reconstruction resolution to match the highest in‐plane resolution of the ^129^Xe images in this study. Additionally, the FLORET acquisition method enabled more efficient k‐space sampling than conventional stack‐of‐stars acquisition, as expected according to Willmering et al.,^2^ which reduces scan time and offers more respiratory bins to be resolved. Although current 3D PREFUL studies have shown feasibility in single‐center, single‐vendor settings, our findings demonstrate the translatability of 3D PREFUL to multiple vendors, thereby making the technique accessible to more centers.

Across all study participants, a strong correlation was observed between VDP values derived from the proposed 3D PREFUL FLORET UTE algorithm and ^129^Xe MRI. The strongest correlation was found for the VDP of the static RVent parameter (VDP_RVent_) and VDP of ^129^Xe MRI (VDP_Xe_). Recent publications have observed similar correlations for the 3D PREFUL technique with both stack‐of‐stars acquisition^33^ and 3D radial UTE acquisition.^34^

Notably, a significant bias (of 10%) toward higher VDP_RVent_ values was observed in comparison to VDP_Xe_ values. Furthermore, the mean difference of VDP measures between 3D PREFUL FLORET UTE and ^129^Xe MRI increased with ventilation defect severity. Several explanations exist for the different VDP values between VDP_RVent_ from 3D PREFUL FLORET UTE and the VDP_Xe_ from ^129^Xe MRI. First, the 3D PREFUL proton ventilation surrogates were derived indirectly from parenchymal‐density changes, whereas ^129^Xe MRI provides direct ventilation measurements derived from the density of inhaled magnetized xenon gas. Second, there were differences in spatial resolution (3 × 3 × 3 mm^3^ for 3D PREFUL FLORET versus anisotropic 3 × 3 × 15 mm^3^ for ^129^Xe MRI). McAllister et al. demonstrated that low‐resolution ^129^Xe ventilation images (voxel size of 2.2 × 6.4 × 21.0 mm^3^) tend to underestimate VDP due to their susceptibility to partial volume artifacts.^35^ In accordance, VDP values of static RVent derived by 3D PREFUL FLORET UTE consistently exceeded VDP values of ^129^Xe. Validity could be improved by comparing 3D PREFUL FLORET data against ^129^Xe ventilation data acquired at the same resolution (e.g., as demonstrated in high‐resolution breath‐hold studies^36^, ^37^) or against computed tomography or V/Q scans. Third, each technique used different thresholds for VDP calculation based on previous studies with different study populations, which could potentially influence our results. While conducting this study, we aimed to use already established thresholds for ^129^Xe as well as 3D PREFUL MRI. Nevertheless, better agreement might be achieved by harmonizing the thresholding techniques. A fourth potential source of difference between 3D PREFUL FLORET UTE and ^129^Xe could be the variation in lung volumes used for image acquisition. The proton images are reconstructed at multiple respiratory phases during tidal breathing, typically starting at functional residual capacity (FRC) plus a mean tidal volume of 0.5 L, a value reported in the literature for men and women.^38^ In contrast, ^129^Xe images may represent a larger lung volume, such as FRC plus 1 L. Hughes et al. reported a trend toward increased ventilation inhomogeneities at higher lung volumes, suggesting that this difference in lung volumes may contribute to variations observed between the two methods.^39^ Fifth, differences in VDP values may arise from segmentation errors of the lung parenchyma and inaccuracies in image registration during the PREFUL ventilation analysis. A final source of the VDP_Xe_ and VDP_RVent_ discrepancy could be related to lung atelectasis, as all ^129^Xe images were acquired first, about 30–45 min before FLORET UTE images. It is possible that the prolonged time in the supine position could have impeded the regional ventilation function preferentially in the FLORET UTE acquisition.

### 4.1 Limitations

The proposed algorithm has several limitations. First, the reconstruction time for temporally resolved images is 1–2 h, which limits the feasibility of real‐time reconstruction on the scanner. However, this is largely restricted by the ANTs software package, which currently does not support GPU processing. This also lengthens the duration for the PREFUL postprocessing, which can take up to 4 h. Future registration software improvements will speed up both reconstruction and postprocessing.

Second, although ^129^Xe imaging is a static breath‐hold measure, 3D PREFUL FLORET UTE provides dynamic measurement during free tidal breathing. Therefore, in ^129^Xe imaging, areas where gas flow is obstructed are depicted as defects, whereas in 3D PREFUL FLORET UTE, defect areas are correlated to areas of decreased tissue compliance or abnormal ventilation filling throughout the breathing cycle. Consequently, future work will explore advantages and disadvantages of each approach at detecting static and dynamic changes in lung pathophysiology.

Third, compared with proton‐based 2D measurements, the main disadvantage of the FLORET UTE acquisition is the lack of perfusion‐weighted information. This issue arises because the inflow effect of fresh blood cannot be measured due to the absence of slice selection. Finally, the clinical importance and repeatability of the dynamic ventilation parameters should be studied in greater detail. It is not yet known how dynamic lung abnormalities vary between diseases under the scope of this 3D PREFUL FLORET UTE technique. Additionally, the correlations presented here could be validated further by comparing against pulmonary function testing data such as forced expiratory volume in 1 s, which has shown strong correlations in 2D^40^, ^41^, ^42^ and 3D^11^, ^30^ but was not included in our study. However, this proof‐of‐concept work highlights that PREFUL‐derived ventilation maps at multiple time points throughout the breathing cycle can be a useful addition in understanding dynamic lung abnormalities and improving clinical outcomes.

## 5 CONCLUSION

Using the optimized binning‐reconstruction and image‐reconstruction algorithm, 3D PREFUL FLORET UTE MRI offers improved isotropic spatial resolution of 3mm^3^ as a result of a more efficient acquisition scheme compared with previous methods. Subsequently, the proposed algorithm demonstrated promising visual correspondence of ventilation surrogate markers with ^129^Xe MRI along with a strong correlation of VDP values and less bias for the dynamic 3D PREFUL FLORET UTE measures. Future work should focus on assessing the repeatability of the proposed method and studying disease‐specific abnormalities.

## CONFLICT OF INTEREST

F.K., A.V., and J.V.C. are shareholders of BioVisioneers GmbH, a company that has interest in pulmonary MRI methods. M.W. and L.W. have received consulting fees from Polarean Imaging, PLC.

## Data Availability

Reconstruction scripts (*Python*) and sample data are located at https://github.com/joeyplum/spatio‐temporal‐recon. We kindly encourage users to ask questions, post issues, and collaborate with us on this repository.
